# A pilot radiometabolomics integration study for the characterization of renal oncocytic neoplasia

**DOI:** 10.1038/s41598-023-39809-9

**Published:** 2023-08-03

**Authors:** Michail E. Klontzas, Emmanouil Koltsakis, Georgios Kalarakis, Kiril Trpkov, Thomas Papathomas, Na Sun, Axel Walch, Apostolos H. Karantanas, Antonios Tzortzakakis

**Affiliations:** 1 Department of Medical Imaging, University Hospital of Heraklion, Crete, Heraklion, Greece; 2 Computational BioMedicine Laboratory, Institute of Computer Science, Foundation for Research and Technology (FORTH), Crete, Heraklion, Greece; 3 Department of Radiology, School of Medicine, University of Crete, Voutes Campus, Heraklion, Greece; 4 Division of Radiology, Department for Clinical Science, Intervention and Technology (CLINTEC), Karolinska Institutet, Stockholm, Sweden; 5 Department of Diagnostic Radiology, Karolinska University Hospital, Solna, Stockholm, Sweden; 6 Department of Diagnostic Radiology, Karolinska University Hospital, Huddinge, Stockholm, Sweden; 7 University of Crete, School of Medicine, 71500 Heraklion, Greece; 8 Department of Pathology and Laboratory Medicine, Alberta Precision Labs, Cumming School of Medicine, University of Calgary, Calgary, Canada; 9 Institute of Metabolism and Systems Research, University of Birmingham, Birmingham, UK; 10 Department of Clinical Pathology, Vestre Viken Hospital Trust, Drammen, Norway; 11 Research Unit Analytical Pathology, German Research Center for Environmental Health, Helmholtz Zentrum München, Neuherberg, Germany; 12 Medical Radiation Physics and Nuclear Medicine, Section for Nuclear Medicine, Karolinska University Hospital, Huddinge, C2:74, 14 186 Stockholm, Sweden

**Keywords:** Renal cell carcinoma, Predictive markers

## Abstract

Differentiating benign renal oncocytic tumors and malignant renal cell carcinoma (RCC) on imaging and histopathology is a critical problem that presents an everyday clinical challenge. This manuscript aims to demonstrate a novel methodology integrating metabolomics with radiomics features (RF) to differentiate between benign oncocytic neoplasia and malignant renal tumors. For this purpose, *thirty-three* renal tumors (14 renal oncocytic tumors and 19 RCC) were prospectively collected and histopathologically characterised. Matrix-assisted laser desorption/ionisation mass spectrometry imaging (MALDI-MSI) was used to extract metabolomics data, while RF were extracted from CT scans of the same tumors. Statistical integration was used to generate multilevel network communities of -omics features. Metabolites and RF critical for the differentiation between the two groups (delta centrality > 0.1) were used for pathway enrichment analysis and machine learning classifier (XGboost) development. Receiver operating characteristics (ROC) curves and areas under the curve (AUC) were used to assess classifier performance. Radiometabolomics analysis demonstrated differential network node configuration between benign and malignant renal tumors. Fourteen nodes (6 RF and 8 metabolites) were crucial in distinguishing between the two groups. The combined radiometabolomics model achieved an AUC of 86.4%, whereas metabolomics-only and radiomics-only classifiers achieved AUC of 72.7% and 68.2%, respectively. Analysis of significant metabolite nodes identified three distinct tumour clusters (malignant, benign, and mixed) and differentially enriched metabolic pathways. In conclusion, radiometabolomics integration has been presented as an approach to evaluate disease entities. In our case study, the method identified RF and metabolites important in differentiating between benign oncocytic neoplasia and malignant renal tumors, highlighting pathways differentially expressed between the two groups. Key metabolites and RF identified by radiometabolomics can be used to improve the identification and differentiation between renal neoplasms.

## Introduction

Renal cell carcinoma (RCC) represents approximately 2% of tumors in adults^1^. Differentiating benign from malignant renal neoplasia is a critical clinical problem in the evolving landscape of renal neoplasia^2,3^. Modern examination methods have been applied to solve this differential dilemma because several oncocytic tumors may share common imaging and morphologic characteristics with RCC^4,5^, potentially resulting in surgical overtreatment of benign kidney tumors. For example, angiomyolipoma (AML) and renal oncocytoma (RO), both considered benign renal neoplasms, contribute to 10% of unnecessary nephrectomies due to the overlapping imaging and morphologic characteristics with RCC^2,6–8^. The general category of “oncocytic renal neoplasia” includes tumors such as benign renal oncocytoma (RO), a newly recognised low-grade oncocytic tumour (LOT)^2,8,9^, an eosinophilic variant of chromophobe RCC (chRCC), as well as tumors sharing common characteristics between RO and chRCC, known as hybrid oncocytic-chromophobe tumour (HOCT), typically seen in hereditary syndromes, such as Birt Hogg-Dubé (BHD)^10^. Some tumors in the last “hybrid” category, particularly if sporadic, generally include mostly indolent with or low malignant potential renal neoplasms^11^. The percentage of renal oncocytic tumors pre-operatively misclassified as malignant has been reported to be up to 27%^12^. Imaging can assist in the differential diagnosis between renal oncocytic tumors and RCC but traditional MRI or multiphasic CT imaging does not provide a definite diagnosis in most cases, even in the eyes of experienced urogenital radiologists^13–15^. Therefore, in order to optimize the differentiation between the two types of lesions, -omics analyses have been employed including metabolomics^16,17^, transcriptomics^18^, and radiomics^19–22^ with variable success. As highlighted in a systematic review and meta-analysis of radiomics/AI methods for RCC diagnosis, the results of existing radiomics manuscripts do not offer a clear advantage in diagnosis compared to human reader evaluation^19^. Since LOT is a newly recognised entity the majority of studies have disregarded LOT^23–30^. In these studies, radiomics analysis has been shown to have a pooled sensitivity and specificity of 83% and 92% respectively for the differentiation between RO and RCC, according to a meta-analysis of all relevant studies^31^. Nonetheless, given the rapidly changing landscape in the pathological diagnosis of renal tumors and the recognition of LOT and HOCT as important entities^8^, results that have been published with outdated pathological classifications of renal tumors need to be taken with caution.

Integrating biological and image-based -omics has been introduced by combining transcriptomics, proteomics, and genomics with radiomics data. Such radiotranscriptomics^32,33^, radioproteomics^34^, and radiogenomics^35–38^ signatures have provided novel biomarkers for the detection of cardiac disease, prediction of cancer aggressiveness, and the distinction between tumour subtypes, as well as for image-assisted distinction between molecular subtypes of tumors. The utilisation of global -omics signatures provides a holistic analysis of the layers of cellular function (genome, proteome, transcriptome)^39^, and a comprehensive characterisation of imaging appearances of a lesion (radiomics)^40^. Such an approach enables the identification of correlates between biological processes and computed tomography (CT) or magnetic resonance (MR) imaging appearances of a specific lesion.

**Figure 1:**
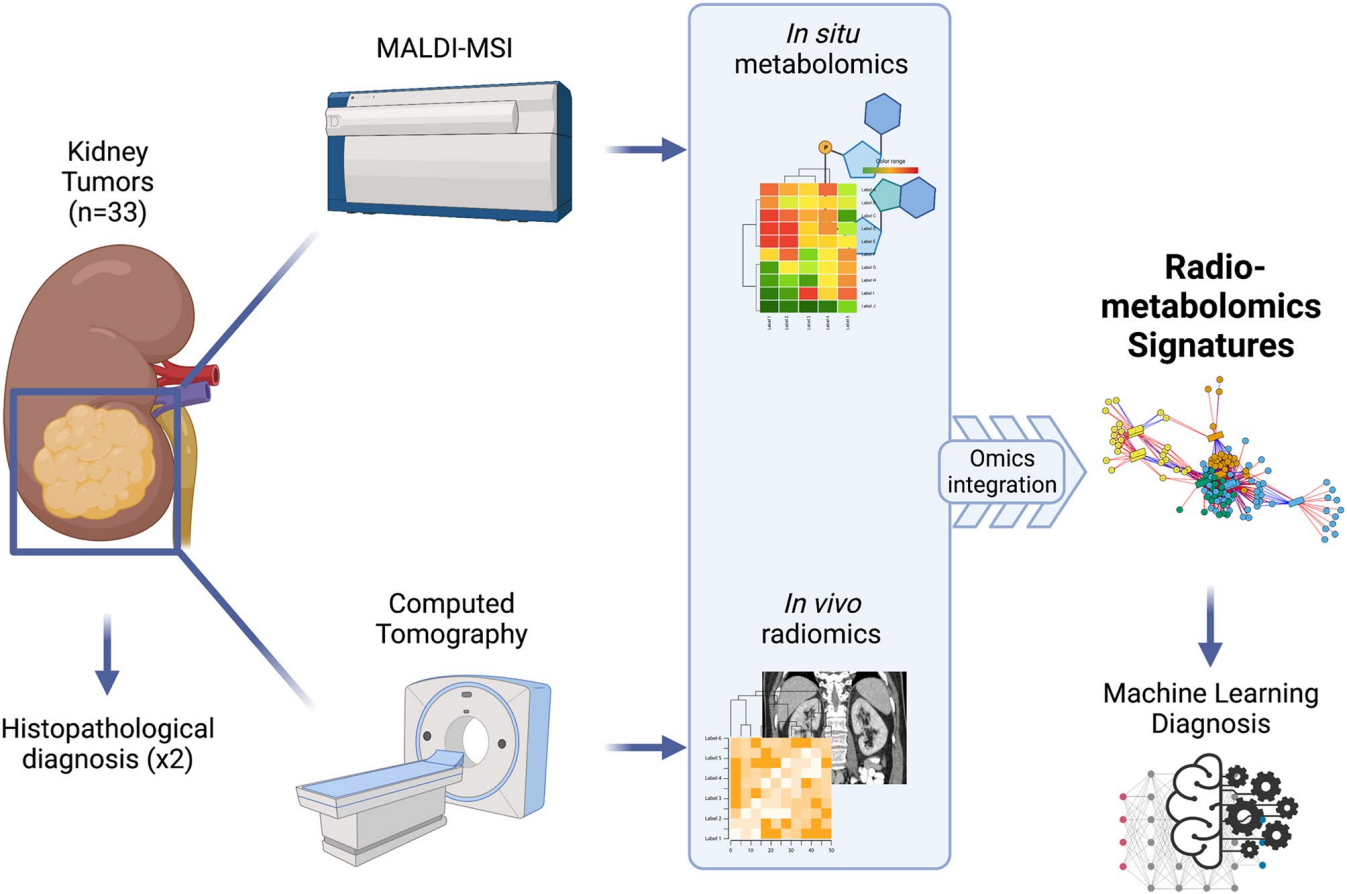
Flowchart describing the radiometabolomics integration pipeline (created with BioRender.com).

To our knowledge, no integration between metabolic and radiomics signatures has been previously published. In this pilot study, we aimed to present a methodology for the integration of metabolomics and radiomics data using as a case study the evaluation of renal tumors, in an attempt develop novel radiometabolomics signatures highlighting the correlations between metabolic and imaging phenotypes of various renal neoplasms. We performed a global radiometabolomics analysis of a well-characterised tumour cohort to identify novel potential biomarkers (metabolites and radiomics features) that may play an important role in determining a malignant versus benign phenotype (Fig. 1). Ultimately, we sought to demonstrate how a radiometabolomics-based machine learning classifier can distinguish between benign renal oncocytic tumors (RO, LOT and HOCT) and malignant RCC types.

## Methods

### Patient recruitment and ground truth establishment

A cohort of 33 renal tumors from n = 28 patients (22 male and 6 female) with an average age of 65.3 years (range 45–87 years) were included prospectively included in this study between September 2016 and September 2018, following the Declaration of Helsinki and approved by the Karolinska University Hospital (Huddinge) Regional Ethical Review Board and Radiation Safety Committee (2018/1626). All patients recruited in the study provided an informed consent. Renal tumors in this study represent the same cohort we used to perform in situ metabolomic analysis previously^17^ and are part of the larger MIDOR cohort that has been used to extract quantitative imaging data for other (to date unpublished) projects. To establish the ground truth diagnosis used in further analyses, all histology specimens were analysed in a blinded fashion by two subspecialist histopathologists. Haematoxylin and eosin (H-E) slides and routine immunohistochemistry stains were utilised to establish a final diagnosis, using the contemporary criteria that were subsequently used as the ground truth for metabolomics, radiomics and machine learning analysis. The study sample included 14 renal oncocytic tumors (“*benign oncocytic renal tumour group*”): 9 RO, 3 HOCT, 2 LOT; and 19 RCC (“*malignant renal tumour group*”) 6 clear cell RCCs (ccRCC), 8 papillary RCCs (pRCC), 4 chRCC, (3 classic chRCC and 1 eosinophilic chRCC), and 1 oncocytic unclassified tumour^2,9,41,42^. Of note, the 3 HOCT cases were from one female patient with verified BHD syndrome.

### CT image acquisition and radiomics data extraction

CT examinations of all patients were retrieved from the institutional RIS-PACS system for^43^ radiomics analysis. Examinations were performed in multiple CT scanners, and a minimum of 64-slices and images were obtained with spiral acquisition at 120 kV at the portal venous phase, with an average slice thickness of 4 mm. Voxel size was resized to 1 × 1 × 4 mm, and a uniform bin width of 32 was used across examinations to ensure robust radiomics feature calculation. Data was subjected to z-score normalization prior to radiomics analysis, to ensure feature reproducibility^43,44^.

Two senior radiology residents (GK, EK) with 5 years of experience in segmentation, manually segmented all tumors using 3D Slicer v4.11.20 (https://slicer.org), blinded to the final diagnosis. Radiomics features were extracted using the PyRadiomics module^45^ of 3DSlicer, and the coefficient of variation (CV) of each radiomics feature was calculated between the two readers. Only features with a CV ≤ 10% were used in further analysis to eliminate variability related to manual segmentation. A total of 944 radiomics features were initially extracted from each tumour; 700 were found stable across multiple segmentations and used for further analysis. These included first-order features, grey level co-occurrence matrix (GLCM), grey level run length matrix (GLRLM), grey level dependence matrix (GLDM), neighbouring grey-tone difference matrix (NGTDM), grey level size zone matrix (GLSZM), 2D and 3D shape features as well as their Laplacian of Gaussian (LoG) and wavelet transformations.

### Metabolomics data acquisition

To investigate in situ metabolomic status, tumour samples from biopsy and resection specimens were arranged in a tissue microarray analysis (TMA) using a semiautomated tissue arrayer MiniCore, as previously described^17^. In brief, representative areas were selected for each case and were marked on H-E-stained slides. We used three cores for resection specimens and usually one core for biopsies, with a diameter of 1 mm. These were extracted from the “donor” block and were arrayed in the “recipient” paraffin block. To validate the data, we used part of a previously published cohort comprising 117 tumors: 59 ROs and 58 chRCCs. Sections of 4 μm were subsequently cut from the TMA blocks.

Matrix-assisted laser desorption/ionisation (MALDI) mass spectrometry imaging (MSI) analysis was performed at the Research Unit Analytical Pathology (Helmholtz Zentrum München), as described previously by Ly et al.^46^. MALDI time of flight (TOF) MSI measurements were carried out on an Ultraflex III MALDI-TOF/TOF MS (Bruker Daltonic, Bremen, Germany) with 60 μm lateral resolution over the analysed mass range of m/z 100–1000 in the negative reflector ion mode. A Smartbeam-II Nd:YAG laser was equipped with a frequency of 100 Hz. The sampling rate of 2.0 GS/s and 200 laser shots were used for each measurement position. MALDI Fourier transforms ion cyclotron resonance (FT-ICR) MSI measurements were performed on a Bruker Solarix 7T FT-ICR-MS (Bruker Daltonic, Bremen, Germany) over the mass range of m/z 50–1000 in the negative ion mode. For each measurement position, 100 laser shots were accumulated using a Smartbeam-II Nd:YAG (355 nm) laser operating at a frequency of 500 Hz.

Following MALDI MSI analysis, the matrix was removed with 70% ethanol and stained with H-E using a fully automated tissue stainer (Tissue Stainer TST 44C; MEDITE, Leica, Nussloch, Germany). Slides were scanned using a MIRAX DESK digital slide-scanning system (Carl Zeiss MicroImaging, Gottingen, Germany). To spatially relate the signal intensities to histopathological features of individual tissue spots, digital images were coregistered to respective MSI data using FlexImaging v. 4.2 and SCiLS Lab version 2017 (Bruker Daltonic, Bremen, Germany). Only the signals that were co-localised with the neoplastic cells were classified.

### Radiometabolomics data integration

Integration of radiomics and metabolomics data was performed systematically in a data-driven manner, as described by Uppal et al.^47^, using the xMWAS package as previously described^47,48^. xMWAS combines Partial Least Squares (PLS), sparse PLS and multilevel sparse PLS methods to achieve systematic integration between any type of -omics datasets. This approach identifies associations between metabolomics and radiomics data, enabling network visualisation and characterising multilevel network communities of functionally/conceptually related features in each dataset. Importantly, it allows the characterisation of network node importance (i.e., the importance of unique metabolites or radiomics features) at each examined condition using the eigenvector centrality metric and enables the identification of nodes essential for the differentiation between two conditions (e.g., RO/LOT/HOCT vs RCC), based on delta centrality measurements. Ultimately, data-driven integration leads to identifying features in each dataset that may play an important role in determining a phenotype of multiple phenotypic layers (e.g., metabolism and gross image appearance on CT) rather than using a single -omics approach. xMWAS analysis was performed in R Studio v 2022.02.3 with R v.4.03 (https://www.R-project.org/). Integration network nodes (metabolites or radiomics features) with a Delta Centrality (Eigenvector centrality_benign_−Eignenvector centrality_malignant_) > 0.1 were considered essential for the differentiation between renal oncocytic and RCC tumors. Metabolite pathway enrichment analysis was performed on nodes highlighted as necessary by radiometabolomics integration using MetaboAnalyst v 5.0^49^.

### Machine learning model development and statistical analysis

Radiomics features and metabolite compounds identified as important by radiometabolomics integration were used to develop a machine learning model to differentiate between benign renal oncocytic tumors and malignant RCC. To reduce machine learning bias related to a class imbalance between benign oncocytic renal tumors and malignant RCC, the Synthetic Minority Oversampling Technique (SMOTE) was used as previously described^37,50^ to produce a final dataset with 20 benign and 20 malignant lesions. Machine learning classifiers were built using an advanced gradient boosting XGboost model implemented in the ‘xgboost’ R package with a linear kernel. Data were split 60:40 in the training sessions; in the validation sets, a random seed and model hyperparameter optimisation was performed with random search (n = 1000 rounds), using a ten-fold cross-validation in the training step. Hyperparameter tuning was performed by random search generating 1000 consecutive random models which yielded the following hyperparameters: eta = 0.104, gamma = 0, max_depth = 6, min_child_weight = 5.31, subsample = 0.654, colsample_bytree = 0.564. Model overfitting was avoided by monitoring the model's accuracy, loss, and early stopping. Model performance in the validation set was assessed using the Area Under the Curve (AUC) metric. The corresponding 95% Confidence Interval was calculated with bootstrapping (n = 2000 rounds) using the pROC R package. For comparison purposes a Support Vector Machines and a Random Forests classifier were build for the combined radiometabolomics datasets using the “e1071” and “randomForests” packages in R. Sensitivity, specificity, positive and negative predictive values and the receiver operating characteristics (ROC) curves were calculated for XGboost models containing all nodes (metabolites and radiomics features) with Delta Centrality (DC) > 0.1, metabolites with DC > 0.1, or radiomics features with DC > 0.1. Differences between ROC curves were compared with DeLong’s method^51^. Statistical significance was defined with a P-value less than 0.05.

## Results

### Radiometabolomics integration

**Figure 2:**
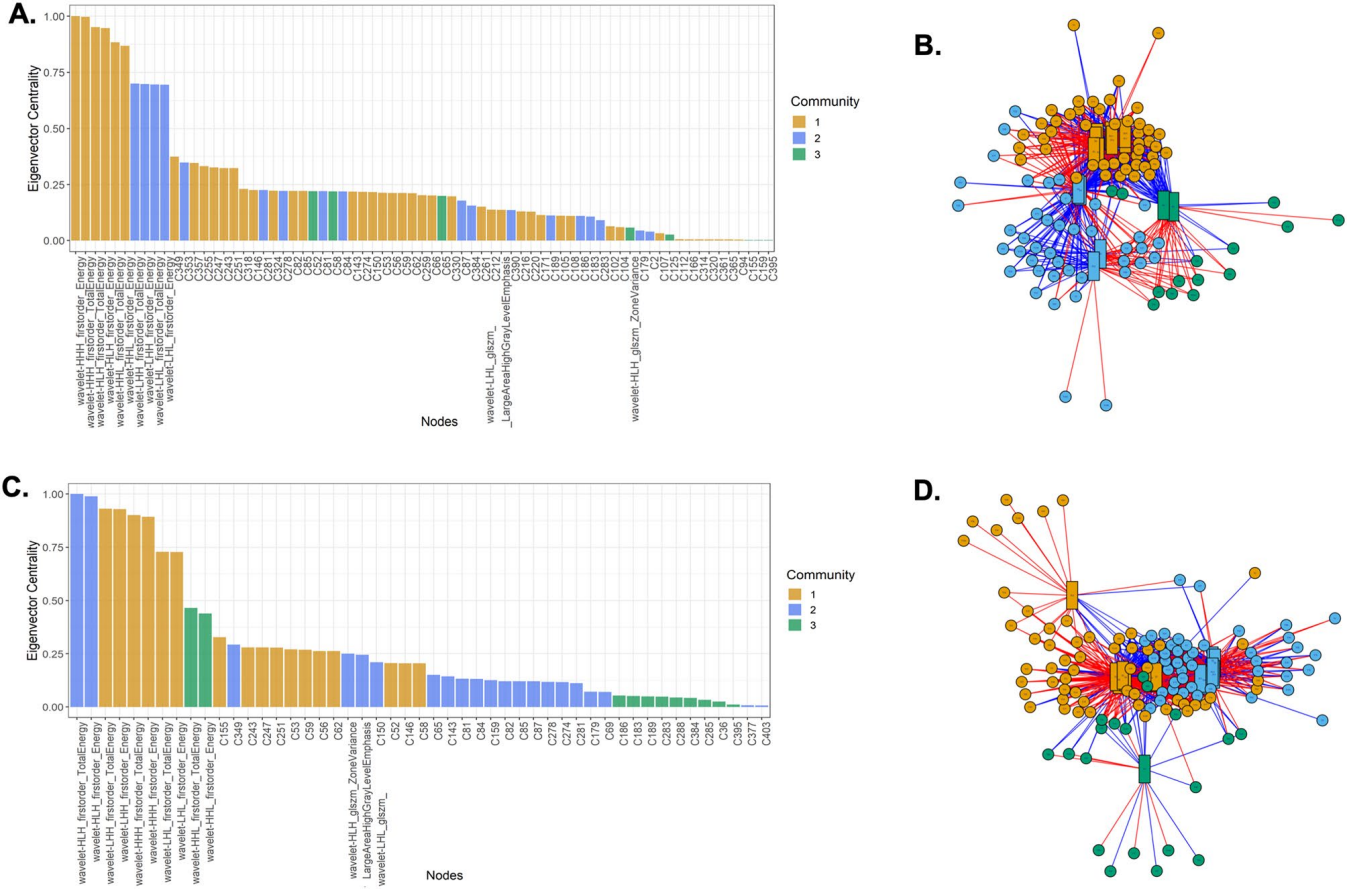
Multilevel detection of metabolite–radiomics feature communities in (**A**, **B**) malignant and (**C**, **D**) benign renal tumours. Eigenvector centrality graphs denote nodes (radiomics features, metabolites) that play a crucial role in maintaining the malignant (**A**) or benign (**C**) phenotype. The respective multi-level community networks demonstrate different node configurations in each group. Radiomics features are indicated by square-shaped nodes, while metabolites are indicated by circular nodes in multi-level community networks (**B**, **D**). Different colours indicate membership in distinct communities. Eigenvector centrality indicates the importance of each radiomics feature and metabolite in network formation. Radiomics features and metabolites with nonzero eigenvector values are displayed.

**Figure 3:**
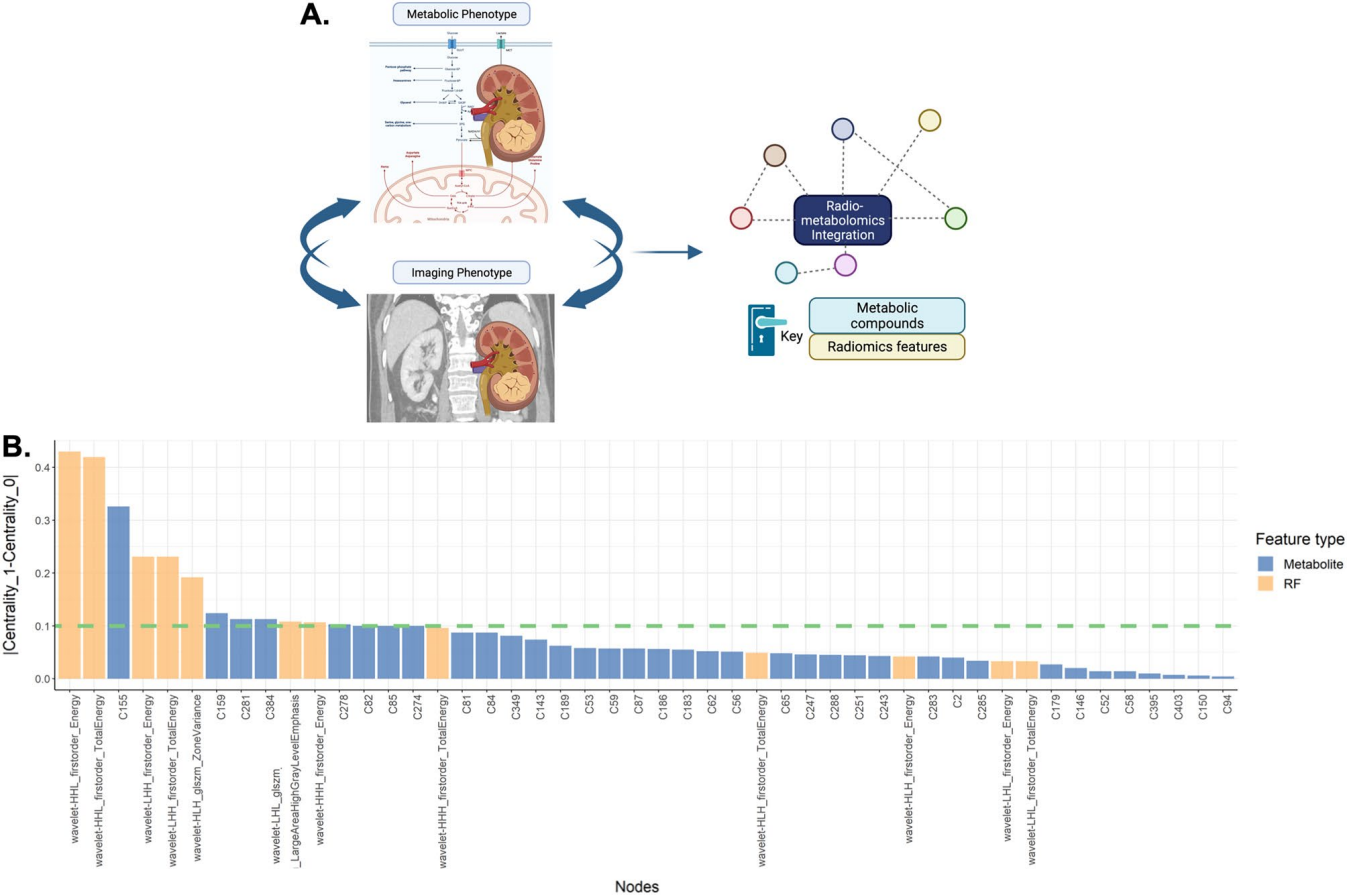
Integration of radiomics and metabolomics data reveals novel features crucial for distinguishing between malignant and benign renal tumours. (**A**) Schematic explaining the concept of radiometabolomics data integration (created with BioRender.com). (**B**) Delta centrality for individual metabolites and radiomics features indicates nodes significant for the distinction between the benign and malignant phenotype. Blue and orange bars represent metabolites and radiomics features, respectively, significant in the distinction between malignant and benign renal tumours. The dashed green line denotes DC > 0.1, which is the threshold of significance for node centrality; DC, delta centrality.

A set of 700 radiomics features were integrated with 771 metabolite features from each tumour to identify the molecules pivotal in determining a “benign” or “malignant” renal phenotype. Network integration of the two datasets revealed three distinct node communities (i.e., metabolites and radiomics features) that characterise RCC. Seventy-one nodes were shown to have an eigenvector centrality > 0, indicating an important role in determining a malignant phenotype. These nodes included 12 radiomics features (wavelet transformations of first order and glszm features) and 59 metabolites (Fig. 2A, B). RO, LOT and HOCT tumors demonstrated a different network configuration with 3 distinct node communities, which included 49 nodes (12 wavelet radiomics and 37 metabolite features) (Fig. 2C, D). DC was calculated to identify features that enable differentiation between two tumour categories, and nodes with DC > 0.1 were considered essential in the distinction between the two conditions. Fourteen nodes were found to have a DC > 0.1, including 6 radiomics and 8 metabolite features (Fig. 3). Radiomics nodes found to be significant were characteristically wavelet decompositions of first order and glszm features. These nodes were used for further pathway analysis and machine learning model building.

### Integration-based metabolic pathway analysis

**Figure 4:**
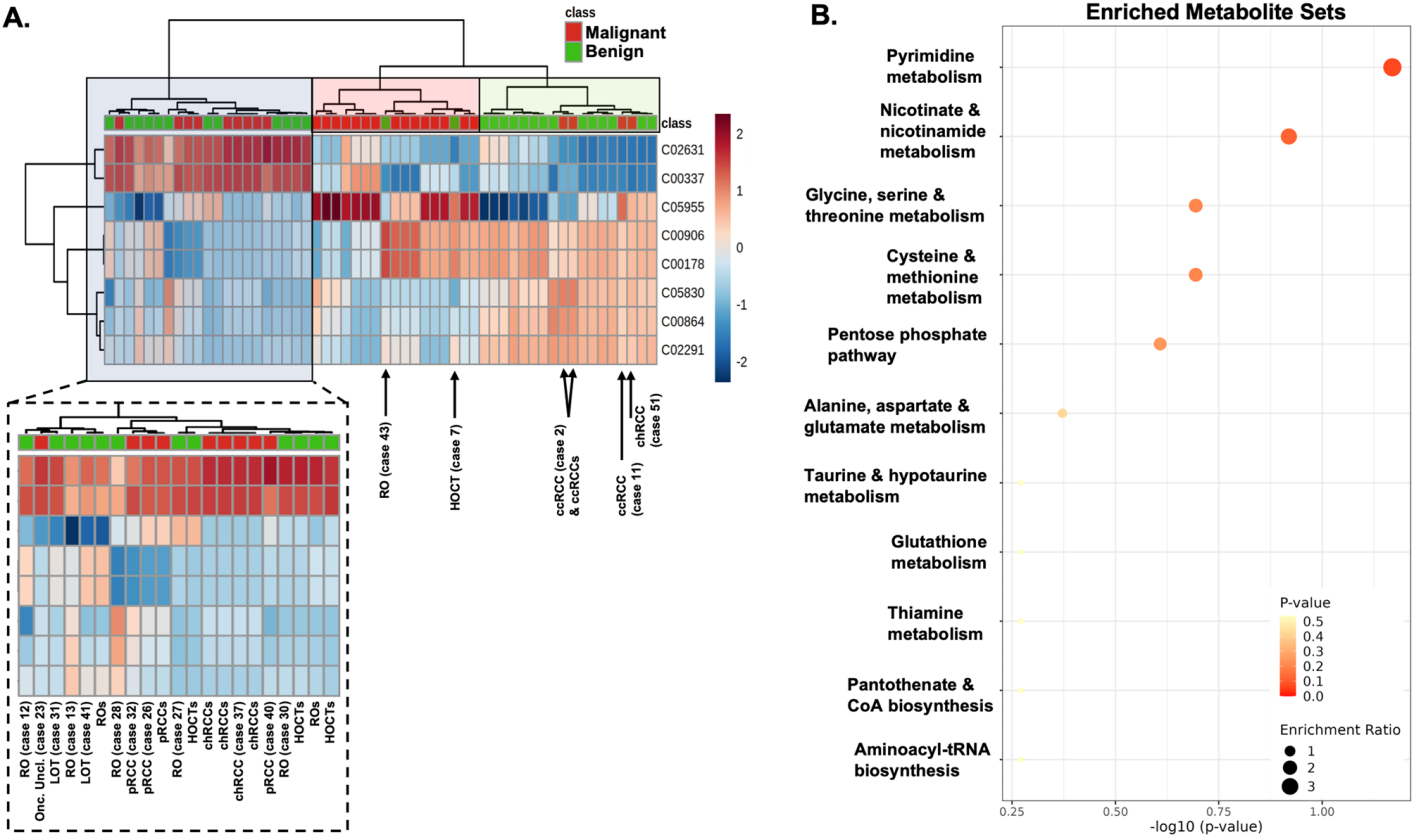
Analysis of metabolite nodes highlighted by radiometabolomics integration. Unsupervised hierarchical clustering of significant nodes (DC > 0.1) (**A**). Columns represent tumour samples, and lines represent metabolite compounds named with the respective KEGG id, clustered with the Euclidean distance metric. Sample clustering revealed three distinct clusters, one predominantly including benign tumours (green shaded), a second predominantly including malignant tumours (red shaded) and a third including a combination of malignant and benign tumours (blue shaded). The zoomed-in insert provides an insight into the tumour types included in the mixed cluster. Metabolic pathway enrichment analysis of the top 25 enriched metabolic pathways (**B**). Red colour intensity indicates the significance of enrichment, whereas the dot size indicates the enrichment ratio. RO, renal oncocytoma; ccRCC, clear cell renal cell carcinoma; pRCC, papillary renal cell carcinoma; chRCC, chromophobe renal cell carcinoma; LOT, low-grade oncocytic tumour; HOCT, hybrid oncocytic/chromophobe tumour; Onc. Uncl., Oncocytic unclassified tumour. Suffixes s stands for synthetic tumour.

Significant metabolites highlighted by radiometabolomics integration were analysed to identify differentially affected pathways in both categories, which correlated to the tumors' imaging (radiomics) phenotype. Unsupervised clustering of these 8 metabolites revealed three major clusters, one containing majority of malignant tumors (Fig. 4A—red shaded dendrogram), a second containing the majority of benign tumors (Fig. 4A—green shaded dendrogram), and a third cluster with a combination of benign and malignant tumors (Fig. 4A—blue shaded dendrogram and zoomed insert). Metabolic pathway analysis revealed that the top-5-enriched pathways differentially expressed in the two groups included: pyrimidine metabolism, nicotinate metabolism, glycine-serine-threonine metabolism, cysteine-methionine metabolism, and pentose phosphate pathway. In addition, other pathways differentially expressed between the two groups included: aminoacid-related pathways such as alanine-aspartate-glutamine, taurine-hypotaurine, glutathione and thiamine metabolism, pantothenate and CoA pathway, and aminonacyl-tRNA biosynthesis (Fig. 4B).

### Machine learning model performance

**Figure 5:**
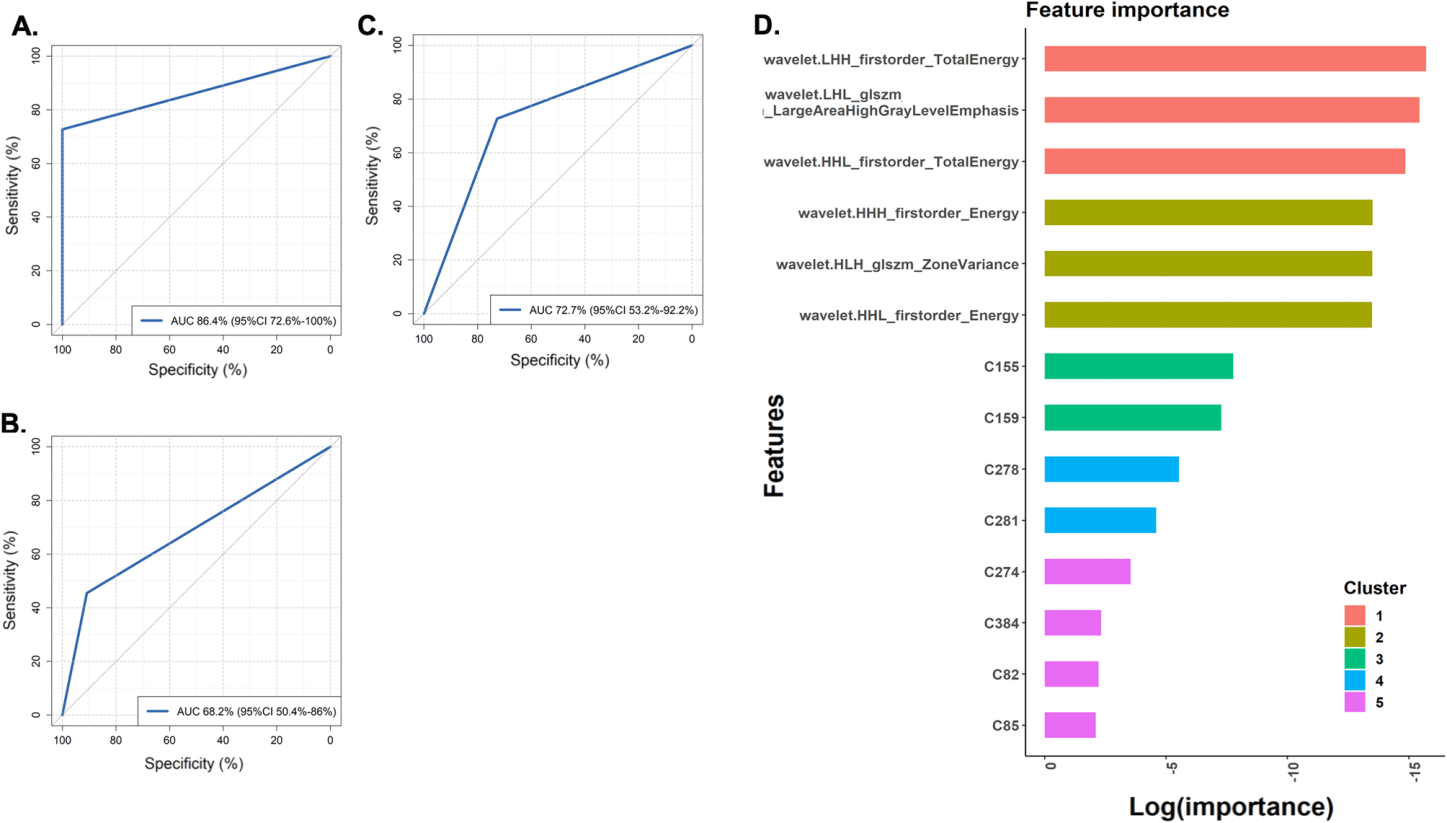
Receiver operating characteristics (ROC) curves for the XGboost classifier developed with radiomics & metabolomics (**A**), radiomics (**B**) and metabolite (**C**) features as identified by radiometabolomics integration analysis. Features with a DC > 0.1 were used for classifier development. Features essential for the classification results of the combined metabolomics & radiomics classifier are presented in (**D**). Features are automatically clustered in five distinct clusters (radiomics vs metabolomics), and the importance of each feature is indicated. AUC, Area Under the Curve; 95%CI, 95% Confidence Interval of the AUC.

Nodes with DC > 0.1 (n = 14) were used for subsequent XGboost model training and validation. A linear kernel XGboost model combining all identified radiomics and metabolomics nodes was built, which achieved an AUC of 86.4% (95% CI from 72.6–100%) (Fig. 5A). Models containing only the radiomics or only the metabolomics nodes achieved significantly lower performance (P < 0.05) with an AUC of 68.2% (95%CI 50.4–86%) for the radiomics-only model and 72.7% (95%CI 53.2–92.2%) for the metabolomics-only model. Sensitivity and specificity values were also different between the radiomics and metabolomics-only models (Figs. 5B, C and Table 1). Both radiomics and metabolomics features were necessary for the performance of the combined XGboost model, with metabolites being more critical than radiomics for the accurate classification into the benign oncocytic (RO/HOCT/LOT) or the malignant RCC group (Fig. 5D). A Support Vector Machines and a Random Forests classifier were also built with radiometabolomics data, demonstrating inferior performance to the XGboost classifier (Supplementary Fig. 1).

**Table 1:** Performance metrics of XGboost classifiers built with integration results: radio-metabolomics, radiomics features or metabolites.

	AUC (%)	Sensitivity (Recall) (%)	Specificity (%)	PPV (Precision) (%)	NPV (%)	F1-score (%)
Radio-metabolomics	86.4	72.7	100	100	78.6	84.2
Radiomics features	68.2	45.5	90.9	83.33	62.5	58.9
Metabolites	72.7	72.7	72.7	72.7	72.7	72.7

AUC, area under the curve; PPV, positive predictive value; NPV, negative predictive value; features with a delta centrality (DC) > 0.1 have been used for classifier development.

## Discussion

This pilot study presents a novel radiometabolomics integration approach that can be used for the evaluation of disease states. The potential value of our approach has been demonstrated in a small-scale case study attempting to differentiate between a group of indolent renal oncocytic tumors (RO, LOT and HOCT) and malignant RCCs. These signatures were used to develop an accurate machine learning classifier to distinguish benign versus malignant tumour phenotypes. They could potentially pave the way for developing novel radiometabolomics-based biomarkers for imaging diagnosis and precision drug targeting.

Differentiating between benign oncocytic renal neoplasms and common RCC types is a challenging task, particularly before the surgical treatment. It has been previously attempted using radiomics^19–22^, metabolomics^17^, ^99m^Tc-Sestamibi SPECT/CT^52^, proteomics^53^, transcriptomics^54^, and genomics^55^. Figure 4A highlights this complex differential of oncocytic neoplasia since 3 HOCT derived from the same BHD patient do not cluster together in the same benign group as expected. Of note is that the two newly recognised benign renal tumors, namely LOT, subcluster in the mixed group of benign and malignant renal tumors. The last-mentioned mixed group contains the only oncocytic unclassified tumour in this cohort, which probably underlines the unclassified nature of this tumour type.

Detection of benign renal tumors using radiomics has achieved AUC that in most cases did not exceed 83% by multiphase CT and well-selected examination conditions, typically based solely on resected surgical specimens^19–22^. The performance of combined radiometabolomics signatures presented herein approached 90%, using only portal phase CT from multiple scanners with a combination of surgical and biopsy specimens. Metabolomics alone could not differentiate between various renal tumour types^17^ and ^99m^Tc-Sestamibi SPECT/CT is an additional tool that may aid the differential diagnosis between RO/HOCT versus RCC^31^. However, ^99m^Tc-Sestamibi SPECT/CT also carries additional radiation exposure, with an effective dose of about 9.5 msV^56^, which is not the case in the currently proposed approach.

Importantly, our strategy highlighted a set of metabolites and pathways that appear to be differentially regulated in oncocytic and RCC tumors. For example, nucleotide (pyrimidine) metabolism significantly differed between the benign renal oncocytic tumors and RCCs. This finding further confirms the network analysis results indicating that purine metabolism is upregulated in RCC^54^. Importantly, nicotinate and nicotinamide metabolism was the second most important differentially regulated pathway between benign oncocytic tumors and malignant RCC. This is in line with current knowledge that nicotinamide *N*-methyltransferase (NNMT) is upregulated in ccRCC and pRCC through the PI3K/Akt/SP1/MMP-2 pathway. This may represent an attempt of the malignant cells to increase acetyl-CoA production for subsequent lipid synthesis by reducing *S*-adenosylmethionine production and suppressing NAD^+^-expensive mitochondrial functions^57^. This is corroborated by our finding that CoA biosynthesis represents one of the key enriched pathways. In fact, drug-targeting of NNMT has been proposed as a promising treatment strategy of ccRCC^58^. Other important pathways that emerged from our radiometabolomics analysis include serine-glycine-threonine metabolism, which plays a role in feeding the folate cycle with one carbon molecule, cysteine and methionine metabolism, and the pentose phosphate pathway. These pathways have been previously implicated in renal cancer metabolism using genomics-proteomics network integration^54^. For example, an upregulation of the pentose phosphate pathway intermediates is characteristic of the renal tumour cells because it feeds the nucleotide synthesis and the energy production through NADPH^59^ Cysteine-methionine biosynthesis, which is also represented in the differentially regulated pathways, is known to play a role in glutathione metabolism, by increasing the capacity of renal cell carcinoma to tolerate oxidative stress^60^. All these differentially regulated pathways illustrate the power of radiometabolomics analysis to identify relevant biomarkers, even in limited samples of relatively rare tumors, while pinpointing potential novel drug targets for cancer treatment.

Wavelet transformations of first order radiomics features also played a vital role in differentiating the two groups. This may be attributed to the imaging features of the neoplastic lesions, such as exophytic margins, line of heterogeneous enhancement, or central stellate scar that may be more common in RO, although it is not specific. Such features include lines and edges, which become more evident when the image undergoes a wavelet transformation^61,62^. In addition, it has been shown that wavelet decomposition of images is not as sensitive to differences arising from a heterogeneous dataset from several CT scanners with variable contrast and noise profiles because they essentially represent bandpass filters that reduce noise effectively^63,64^. Therefore, wavelet decomposition in our dataset could have served as a filter to compensate for the dataset heterogeneity by eliminating external batch effects, highlighting lines and edges, and identifying more robust features for the differential diagnosis between RO and HOCT versus RCC.

Several studies have been published with CT based radiomics analyses of kidney tumors^65^. Some of them have attempted the differentiation between malignant and benign lesions with variable success. One of the biggest studies available, has extracted texture radiomics features from 501 renal tumors achieving an AUC < 65% in distinguishing between benign and malignant ones^28^. Radiomics were better at diagnosing cysts with an AUC reaching 92% in a cohort of 192 patients^66^, however the majority of benign lesions do not have a cystic appearance. This is important since radiomics did not achieve more than 80% AUC for the differentiation between benign and malignant cystic lesions^67^. It is also important to note that most studies consider RO as the only tumor in the benign group which is not in accordance to recent WHO guidelines where RO are grouped together with LOT and HOCT^42^, as done in our study. The results of these studies are therefore, due to different group composition, not comparable to ours. To the best of our knowledge no metabolomics-based machine learning models have been published for the differentiation between benign and malignant renal lesions. Our results confirmed the findings of published literature with a radiomics-only performance of approximately 70%, while presenting a novel methodology where the integration of radiomics and metabolomics increased the performance in differentiating malignant lesions (solid, cystic or mixed) to at least 86%.

Our study has certain strengths and limitations. Strengths include the first demonstration of radio-metabolomics data integration, the establishment of the ground truth by two pathologist subspecialist readings, and using combined biological and imaging data for complete characterisation of the examined tumors. Limitations of our study include the relatively small sample size, comparable to other seminal studies in the literature^20^. However, using high-dimensional data affords high-fidelity tumour characterisation, reducing the need for a larger sample size. Nonetheless, a follow-up study with a bigger sample size is required to confirm the results of our work on RCC. Another limitation is the lack of an external validation dataset for the evaluated machine learning model. However, using a diverse CT dataset from multiple scanners and combining biopsy and surgical samples for metabolomics ensures that the model has been trained to recognise a diverse dataset and is potentially applicable to external data sets.

Everyday clinical diagnostic dilemmas like the accurate preoperative differentiation of renal oncocytic neoplasia versus malignant RCC subtypes lead to continuous research approaches that add value in the specific research field. In summary, in this pilot study, we present a novel radiometabolomics integration and machine learning pipeline. Differentiation between benign renal oncocytic tumors and RCCs has been used to demonstrate potential applications of the method. This resulted in a highly accurate classifier between the two tumour groups, using a combined set of imaging and metabolic biomarkers. An integrated radiometabolomics approach may provide a tool for preoperative diagnostic differentiation between renal neoplasms and highlight relevant target pathways that may be used for future drug development.

### Supplementary Information

Supplementary Figure 1.

## Data Availability

The datasets generated during and/or analysed during the current study are available from the corresponding author on reasonable request.
