# Supplementary material for: A pilot radiometabolomics integration study for the characterization of renal oncocytic neoplasia
Source: Sci Rep. 2023 Aug 3;13:12594. doi: 10.1038/s41598-023-39809-9 (PMC10400617; doi:10.1038/s41598-023-39809-9)
Supplement: Supplementary file 1 — Supplementary Figure 1. [file 41598_2023_39809_MOESM1_ESM.docx]

**
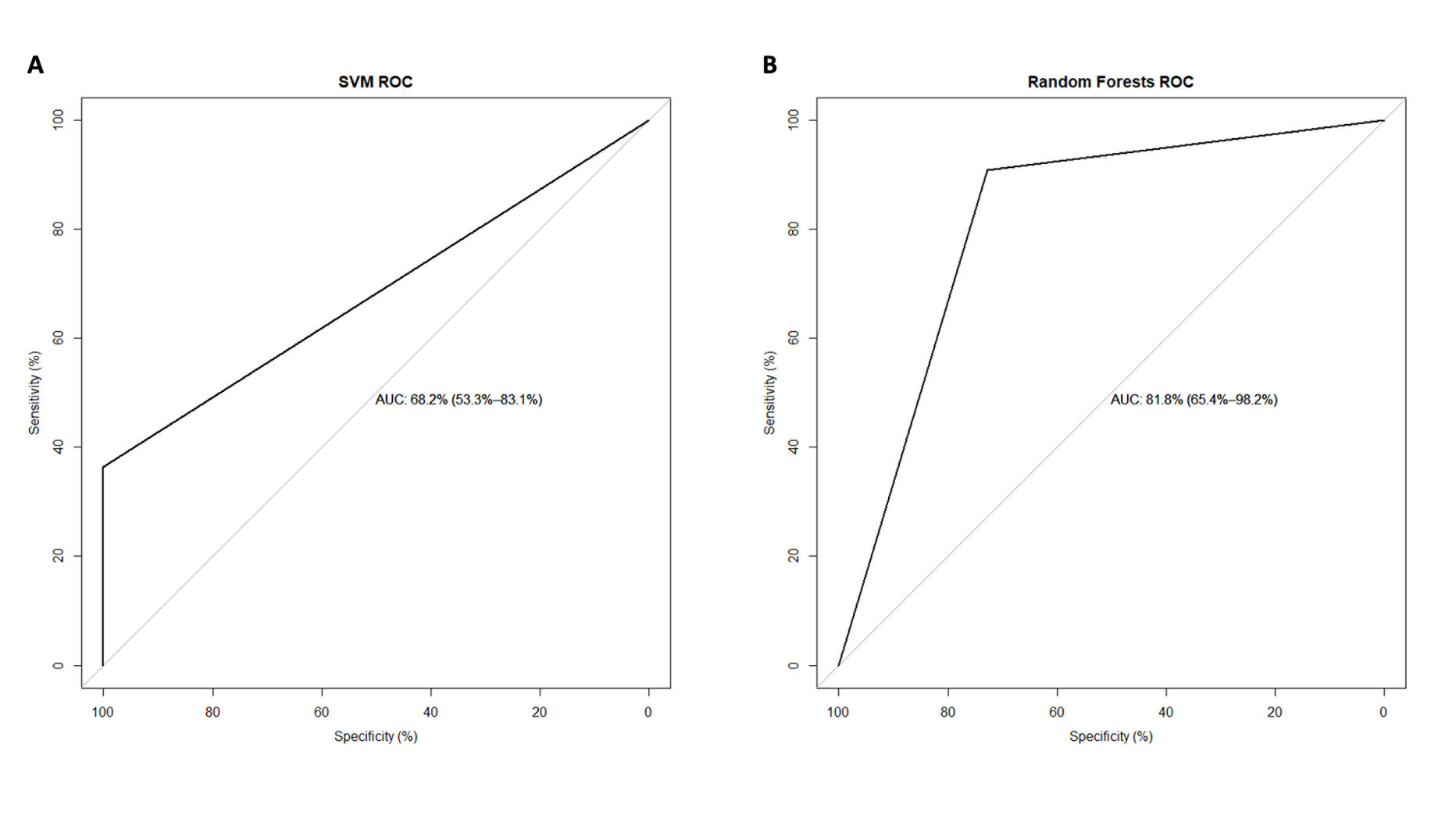
Supplementary Figure 1**

**Supplementary Figure 1**. Receiver operating characteristics (ROC) curves for the Support Vector Machines (A) and Random Forests (B) classifiers created with data identified by radiometabolomics analysis
